# Effects of dietary supplementation with oridonin on the growth performance, relative organ weight, lymphocyte proliferation, and cytokine concentration in broiler chickens

**DOI:** 10.1186/s12917-018-1359-6

**Published:** 2018-01-31

**Authors:** Qiu Jue Wu, Xiao Chuan Zheng, Tian Wang, Tie Ying Zhang

**Affiliations:** 1 College of Animal Science and Technology, Nanjing Agricultural University, No. 6, Tongwei Road, Xuanwu District, Nanjing, 210095 Jiangsu People’s Republic of China; 2 State Key Laboratory of Animal Nutrition, NO. 2, Yuan Ming Yuan West Road, HaiDian District, Beijing, 100193 People’s Republic of China; 3 College of Animal Science and Technology, Henan University of Science and Technology, Luoyang, 471003 Henan People’s Republic of China

**Keywords:** Oridonin, Lymphocyte proliferation, Phagocytisis of neutrophils, Cytokine, Broiler

## Abstract

**Background:**

The present study was conducted to investigate the effects of oridonin (ORI) on growth performance, relative organ weight, lymphocyte proliferation, phagocytic function of neutrophils, and cytokine concentration in broiler chickens. A total of 240 one-day-old Arbor Acres male broilers were randomly assigned to four treatments with six replicate pens of 10 broiler chickens per pen. Broiler chickens were fed diets based on four levels of dietary ORI (0, 50, 80 and 100 mg/kg) for a 42-d feeding trial. The experimental diets were fed in three phases: 1 to 14 d, 15 to 28 d and 29 to 42 d.

**Results:**

The results indicated that ORI has no influence on the growth performance (*P* > 0.05). However, ORI increased the relative weights of spleen and bursa, the number of proliferation peripheral blood T and B lymphocytes, the phagocytic rate of neutrophils, as well as the Interleukin-2 (IL-2), Interleukin-4 (IL-4) and tumor necrosis factor-a (TNF-a) serum concentrations in serum in broilers at days 14, 28 and 42 (*P* < 0.05).

**Conclusions:**

In conclusion, ORI can enhance immune function and resistance to disease in broiler chickens by stimulating T and B lymphocyte formation, division, and proliferation, as well as the modulation of Th1/Th2 cytokine secretion profiles.

## Background

With the rapid development of the poultry industry, high stocking densities and high yield requirements have induced broiler chickens to have higher immunological stress reactions and increased the potential risk of disease outbreaks in commercial poultry flocks. Immunological stress may cause the loss of immune homeostasis and trigger immunological stress responses, followed by enteritis, mortality, welfare problems and economic losses due to production losses and costs for treatment or prevention. Thus, exploring alternative substances and strategies to modulate the broiler immune system is of great significance.

Oridonin (ORI) as a multifunctional compound initially isolated from *Isodon rubescens* (a. k. a. “*Rabdosia rubescens*”, traditional Chinese medicine name “*Donglingcao*”), is now widely used as a drug for humans. Correspondingly, it has been proven to possess considerable anticancer, antioxidation, and anti-inflammatory properties, it has been used to restrain bacterial growth and viral reproduction, detoxify, reduce pain, invigorate the stomach and protect other organs in Asian countries for hundreds of years [1–3].

ORI is considered to be an immune-enhancing dietary supplement, which not only improves phagocytes and inflammatory cytokine production but also exerts immuno-modulating functions for the body. Several studies have provided evidence that ORI is involved in inflammation and antitumor activities [4]. Studies in broilers showed that dietary ORI supplementation decreases expression of the proinflammatory cytokine in the LPS-induced inflammatory response [5]. It has been well documented that the inclusion of oridonin in rat diets can promote the development of immune organs, stimulate organ immunoglobulin secretion, and prohibit the colonization of *Escherichia coli* and *Salmonella*. Additionally, ORI component supplementation has been reported to enhance T lymphocyte responses in vivo [6].

It is well known that immune-modulating nutrients can be harmful if not utilized in moderation. However, whether oridonin could be used as an anti-inflammatory treatment, and the mechanisms underlying its anti-inflammatory activity, remains unknown. Therefore, the following study was conducted to further investigate the effect of dietary supplementation of ORI on growth performance, relative organ weights, lymphocyte proliferation, and cytokine concentration in broiler chickens and to gain a better understanding of the application of dietary ORI supplementation in the poultry industry for improving the health and preventing infectious diseases among broilers.

## Methods

### Animals, study design and diets

A total of 240 one-day-old male broiler chickens (Arbor Acres) were obtained from a commercial hatchery (Broiler Breeder of South Khorasan Complex Productive Co., Iran). All birds were weighed individually and were randomly assigned to four dietary treatment groups in a completely randomized design, each of which included 6 replicates with 10 birds per replicate. The 4 treatment groups were as follows: the control group, in which birds were received the basal diet. The ORI treatment groups, the basal diet was supplemented with oridonin (ORI) at 50 mg/kg, 80 mg/kg or 100 mg/kg (O1, O2 and O3 treatments), respectively. The trial lasted 42 days. Chicks were housed in three-story step cages (2 m × 1.4 m × 0.38 m) in an environmentally controlled room. The rearing room temperature and lighting cycle were provided according to procedure of broiler rearing and management during the period of experiment. The birds were fed the experimental diets in three phases 1 to 14 d, 14 to 28 d and 28 to 42 d. The basal diets were of the maize-soybean-type. The experimental diets were formulated based on the National Research Council (1994) [7] to meet or exceed the nutrient requirements for broiler chickens (Table 1). Fresh diets were prepared once a week and were stored in sealed bags at 4 °C. Feed and water were provided ad libitum throughout the experiment.

**Table 1:** Ingredients and chemical composition of diets used during starter (1–14 d of age), grower (15–28 d of age), and finisher periods (29–42 d of age)

Ingredients (g/kg)	1–14 d	15–28	29–42 d
Corn	428	459	476
Soybean meal (43%, crude protein)	365	250	203
Wheat	130	220	250
Soybean oil	17	8.5	10
Corn gluten meal	20	0	0
Canola meal	0	25.5	25
Na chloride	2.3	3	2.8
Dicalcium phosphate	15	12.5	11
Na bicarbonate	2.4	0.9	1
Ca carbonate	10.8	10.4	11
DL-Methionine	2.7	1.5	1.5
L-Lysine·HCl	2.2	0.6	1.1
Premix^a^	2	2	2
Multi-enzyme	0.3	0.3	0.3
Phytase	0.3	0.3	0.3
Bentonite	0.0	5.5	5
Prebiotics	2	0	0
Total	1, 000	1, 000	1, 000
Calculation of nutrients (g/kg)			
Apparent metabolism energy (MJ/kg)	12.5	12.7	12.9
Crude protein	222	201	193
Calcium	9.7	9.3	9.0
Available phosphorus	4.7	4.5	4.2
Lysine	13.8	11.3	11.0
Methionine	6.0	4.7	4.3
Methionine + cysteine	8.1	7.6	7.1

^a^Premix provided per kg of diet: Vitamin A (transretinyl acetate), 10,000 IU; Vitamin D_3_ (cholecalciferol), 3000 IU; Vitamin E (all-*rac*-*α*-tocopherolacetate), 30 IU; menadione, 1.3 mg; thiamine 2.2 mg; riboflavin, 8 mg; nicotinamide, 40 mg; choline chloride, 600 mg; calcium pantothenate, 10 mg; pyridoxine·HCl, 4 mg; biotin, 0.04 mg; folic acid, 1 mg; vitamin B_12_ (cobalamine), 0.013 mg; Fe (from ferrous sulfate), 80 mg; Cu (from copper sulfate), 8 mg; Mn (from manganese sulfate), 110 mg; Zn (Bacitracin Zn), 65 mg; iodine (from calcium iodate), 1.1 mg; Se (from sodium selenite), 0.3 mg

### Sample collection and procedures

In this experiment, the pen was the experimental unit, and data on body weight and feed intake were measured weekly, those data were used to calculate weight gain (WG), feed intake (FI) and feed conversion ratio (FCR).

At 14, 28 and 42 days of age, twelve broilers from each treatment were used for sample collecting. The birds were weighed, and blood samples were collected and separated by centrifugation at 3000×g for 15 min at 4 °C. Serum samples were frozen at − 80 °C until ELISA analysis. Then, all of the 24 broilers were sacrificed by exsanguination. After decapitation, internal organs (liver, spleen, pancreas, gizzard and bursa) were excised and weights of these organs were measured. Organ indexes were calculated as weight of organ (g)/100 g body weight.

### Measurement of lymphocyte proliferation by the MTT method

Take fresh anticoagulated blood from the wing vein on d 14, 28 and 42 to a heparinized centrifuge tube.The peripheral blood lymphocytes were obtained using lymphocyte separation medium. Then cells were washed, resuspended, seeded and cultured according the MTT method, respectively. Subsequently, the number of proliferation peripheral blood T and B lymphocyte were calculated using the following equation: SI = OD570 (T/B lymphocyte proliferation group)/OD570 (control group).

### Isolation and examination of the phagocytic function of neutrophils

Neutrophils of broiler blood were isolated using the Histopaque (Sigma–Aldrich) separating medium. The cells were centrifuged, withdrawn, washed and detached in plastic tubes, respectively. Then, the pellet were resuspended in the Hank’s medium.

Cells were counted using the phase-contrast microscopy at 40.

Hank’s cellular suspension (5 × 10^5^ neutrophils/mL) were then put into the wells of plastic macrophage migration, and discarded the supernatant. The cells were stained with acridine orange. Neutrophils was phagocytosed and dead bacteria (orange) were determined. The number of particles ingested per 100 neutrophils was expressed as the phagocytosis index (PI). The percentage of cells that had phagocytosed was expressed as the phagocytosis percentage (PP).

### ELISA for the determination of TNF-α, IL-2 and IL-4 production

The serum concentrations of TNF-α, IL-2 and IL-4 were determined using commercial chicken ELISA kits (Adlitteram Diagnostic Laboratories, San Diego, CA, USA).

### Statistical analysis

Variance analyses were done using the General Linear Model procedure of the Statistical Package for Social Sciences 20.0 (SPSS Inc., Chicago, IL, USA) as a completely randomized design. The significant differences among different treatment means were separated using the Duncan’s new multiple range test at *P* ≤ 0.05.

## Results

### Growth performance

All growth performance parameters were not influenced (*P* > 0.05) by dietary ORI levels (Table 2).

**Table 2:** Effects of ORI on the growth performance of broilers

Items	Diet Treatments^a^				SEM^b^	*P*-Value
	CON	O1	O2	O3		
1–14 d						
FI^c^ (g/d)	41.8	41.5	41.4	41.0	0.1	0.20
WG^c^ (g/d)	30.1	30.3	30.4	31.1	0.2	0.37
FCR^c^	1.39	1.37	1.36	1.32	0.02	0.17
14–28 d						
FI^c^ (g/d)	127	126	126	125	1.3	0.24
WG^c^ (g/d)	81.4	82.4	84.0	83.9	1.2	0.10
FCR^c^	1.56	1.53	1.50	1.49	0.04	0.31
28–42 d						
FI^c^ (g/d)	210	209	209	207	1.7	0.19
WG^c^ (g/d)	99.5	100	100.5	101.5	1.0	0.14
FCR^c^	2.11	2.09	2.08	2.04	0.06	0.21
1–42 d						
FI^c^ (g/d)	127	126	125	123	1.0	0.33
WG^c^ (g/d)	69.4	69.2	69.4	69.1	0.9	0.21
FCR^c^	1.83	1.82	1.80	1.78	0.02	0.25

^a^Control = basal diet; O1, O2, and O3 groups = basal diet with 50, 80, and 100 mg/kg ORI, respectively

^b^Standard error of the mean based on pooled estimate of variation

^c^FI = feed intake; WG = weight gain; FCR = feed conversion ratio

### Relative organ weight

Compared with the control, broilers fed basal diet supplemented with ORI (Table 3) significantly increased relative weights of spleen and bursa at day 14, 28 and 42 (*P* < 0.05). However, inclusion of ORI did not have a significant effect on the relative weights of thymus, liver, pancreas or gizzard (*P* > 0.05).

**Table 3:** Effects of ORI on weight of internal organs of broiler chickens (g/100 g body weight of bird)

Items	Diet Treatments^1^				SEM^2^	*P*-Value
	CON	O1	O2	O3		
14 d						
Liver	2.73	2.76	2.74	2.79	0.24	0.333
Spleen	0.08b	0.13a	0.15a	0.14a	0.02	0.123
Pancreas	0.42	0.40	0.38	0.39	0.67	0.231
Gizzard	2.15	2.25	2.34	2.38	0.23	0.164
Bursa	0.12b	0.16a	0.17a	0.17a	0.01	0.109
28 d						
Liver	2.87	2.95	3.02	3.08	0.24	0.233
Spleen	0.10b	0.17a	0.17a	0.18a	0.01	0.03
Pancreas	0.37	0.37	0.36	0.35	0.04	0.671
Gizzard	1.67	1.92	1.73	1.78	0.23	0.254
Bursa	0.14b	0.18a	0.19a	0.20a	0.01	0.214
42 d						
Liver	3.50	3.46	3.35	2.84	0.21	0.27
Spleen	0.12b	0.21a	0.22a	0.23a	0.01	0.03
Pancreas	0.29	0.28	0.26	0.27	0.03	0.150
Gizzard	1.05	1.25	1.20	1.21	0.21	0.124
Bursa	0.16b	0.20b	0.20b	0.21a	0.13	0.104

^1^Control = basal diet; O1, O2, and O3 groups = basal diet with 50, 80, and 100 mg/kg ORI, respectively

^2^Standard error of the mean based on pooled estimate of variation

^a-b^Means within the same row that do not share a common superscript are significantly different (*P* < 0.05); *n* = 8

### Proliferation peripheral blood T and B lymphocyte

As shown in Table 4, inclusion of ORI in the diet significantly increased the number of proliferation peripheral blood T and B lymphocytes at day 14, 28 and 42 when compared with the control group (*P* < 0.05).

**Table 4:** Effects of ORI on the number of proliferation peripheral blood T and B lymphocyte of broiler chickens (%)

Items	Diet Treatments^1^				SEM^2^	*P*-Value
	CON	O1	O2	O3		
T lymphocyte						
14 d	0.13b	0.29a	0.33a	0.36a	0.09	0.031
28 d	0.13b	0.32a	0.36a	0.38a	0.04	0.027
42 d	0.51b	0.71a	0.77a	0.76a	0.11	0.031
B lymphocyte						
14 d	0.13b	0.27a	0.31a	0.37a	0.05	0.043
28 d	0.16b	0.24a	0.22a	0.26a	0.03	0.024
42 d	0.14b	0.47a	0.46a	0.50a	0.02	0.032

^1^Control = basal diet; O1, O2, and O3 groups = basal diet with 50, 80, and 100 mg/kg ORI, respectively

^2^Standard error of the mean based on pooled estimate of variation

^a-b^Means within the same row that do not share a common superscript are significantly different (*P* < 0.05); *n* = 8

### Phagocytic rate and phagocytic index of neutrophil

Dietary ORI supplementation had no significant effect on the phagocytic index of neutrophils on either day 14 or 28 (Table 5). However, dietary ORI supplementation significantly increased the phagocytic index of neutrophils on day 42 (*P* < 0.05). Dietary ORI supplementation significantly increased the phagocytic rate of neutrophils of broilers on days 14, 28 and 42 (*P* < 0.05).

**Table 5:** Effects of ORI on phagocytic rate and phagocytic index of neutrophil of broiler chickens

Items	Diet Treatments^1^				SEM^2^	*P*-Value
	CON	O1	O2	O3		
Phagocytic rate (%)						
14 d	16.56b	21.18a	21.65a	24.61a	0.92	0.042
28 d	25.12b	34.47a	36.27a	39.12a	1.21	0.038
42 d	25.47b	38.37a	40.28a	41.67a	1.82	0.026
Phagocytic index						
14 d	1.38	1.40	1.39	1.42	0.17	0.524
28 d	1.64	1.97	2.04	2.17	0.21	0.178
42 d	1.79b	2.67a	2.85a	2.95a	0.79	0.037

^1^Control = basal diet; O1, O2, and O3 groups = basal diet with 50, 80, and 100 mg/kg ORI, respectively

^2^Standard error of the mean based on pooled estimate of variation

^a-b^Means within the same row that do not share a common superscript are significantly different (*P* < 0.05); *n* = 8

### Cytokine levels in serum

As showen in Table 6, compared with the basal diet, IL-2, IL-4 and TNF-a levels in the serum of broilers were markedly increased by the ORI on day 14, 28 and 42 (*P* < 0.05).

**Table 6:** Effects of ORI on the concentration of serum cytokine of broiler chickens (ng/L)

Items	Diet Treatments^1^				SEM^2^	*P*-Value
	CON	O1	O2	O3		
14 d						
IL-2	11.37b	23.28a	25.49a	30.12a	0.62	0.037
IL-4	96.97b	253.47a	276.37a	337.37a	3.24	0.012
TNF-a	214.77b	456.32a	469.87a	496.78a	4.45	0.027
28 d						
IL-2	12.07b	21.58a	25.34a	29.72a	1.02	0.021
IL-4	103.42b	188.67a	205.67a	211.34a	3.45	0.019
TNF-a	425.38b	675.30a	692.34a	732.04a	6.72	0.039
42 d						
IL-2	14.24b	26.23a	28.37a	28.97a	0.95	0.019
IL-4	114.37b	190.24a	219.37a	286.34a	1.38	0.031
TNF-a	109.37b	385.34a	403.37a	465.35a	7.65	0.047

^1^Control = basal diet; O1, O2, and O3 groups = basal diet with 50, 80, and 100 mg/kg ORI, respectively

^2^Standard error of the mean based on pooled estimate of variation

^a-b^Means within the same row that do not share a common superscript are significantly different (*P* < 0.05); *n* = 8

## Discussion

The literature suggests that oridonin could increase the average daily gain of murine injected with LPS, which was probably ascribed to the anti-inflammatory potential of oridonin by suppressing the production of pro-inflammatory cytokines (Xu et al. 2009) [8]. However, the present study showed that ORI supplementation has no effect on WG, FI or FCR at any recorded ages in the chickens. These results were consistent with results by Zheng et al. (2016) [5], who reported that adding of ORI did not affect the growth performance of broilers. The discrepancies among our findings with other studies for WG and FI are primarily due to animal species.

Several studies have reported that antibacterial activity of oridonin should be regarded as having the promising effect in the healing of gastro-duodenal complaints [9]. Therefore, oridonin can be used as a protective additives of the gastrointestinal system of the body, but may not paly some valuable roles in the growth performance of broilers when broiler chickens are kept in the clean environment and provided the highly digestible diets. Additionally, the dosage and purity of oridonin, breeding density of broilers, and many other factors can be due to inconsistent results.

In our present study, no differences were observed among the ORE treatments in the relative weights of thymus, liver, pancreas or gizzard. However, the relative weights of spleen and bursa were increased in response to feeding of basal diets containing oridonin, which was in line with results of Zheng et al. (2016) [5]. The spleen and bursa are the most important immune organs in the broiler chicken body, and they can suppress immune function by various approaches and induce the status of immunosuppression in broilers. Thus, the measurement of internal immune organ weight is a common method of estimating immune status in chickens [10]. In our present study, the increased immune ability, was likely attributed to the immunosuppressive effect of oridonin [11], may also showed that oridonin can improve immune function in broilers, thus explaining the good health status of broilers in the current study.

T and B lymphocytes play central roles in immune response to any type of stress and/or aggression. Consequently, the number of T and B cells could be a fungible index of lymphocyte immunityability. Moreover, the thymus, spleen and bursa are sites of B and T cell differentiations in broilers. The relative weights of lymphoid organ are used for reflecting the immune status of birds, and changes in weight may be related with alterations in the immune function of lymphoid organs [12]. Because ORI is an immunoenhancer, it encourages faster cell proliferation and differentiation in the immune system of experimental animals. It seems that dietary ORI stimulates the T and B lymphocyte formations, proliferation, and the division, as well as activity of blood. Liu et al. (2007) found that ORI is able to accelerate proliferation of lymphocytes, and that ORI affects body’s immune function and disease resistance [11].

In the present study, we chose ORI as the irritants of T lymphocytes, which can activate many intra-lymphocyte signaling pathways [13]. For the irritants, we observed an increases in immune response in the number of peripheral blood T and B cell counts. This might be associated with ORI’s ability to activate T cells, which may be partially due to T cell depletion in the thymus and the peripheral immune system. Some pharmacological studies show that oridonin is quickly removed from the circulatory system (the level of oridonin in blood was reduced by 78% within 2 h) [14]. The rapid removal of oridonin may explain the different effects of oridonin in vitro and in vivo to some extent. Additional studies will be required before fully understanding its potential applications in the modern broiler breeding.

Neutrophils, as the richest peripheral circulating immune cells, constitute the first-line defense against invading pathogens and act pivotal part in the early stages of inflammation and immune responses. Neutrophil functions, including phagocytosis, degranulation, cytokine release and neutrophil extracellular traps (NETs) formation, play critical roles in the innate immune response against infection through their ability to quickly identify and remove invading germs. Therefore, the phagocytic effect of neutrophils is an important immune parameter because it is strong connections with the clearance of pro-inflammatory necrotic tissue and cell residues.

The results showed that addition of ORI significantly increased the phagocytic index of neutrophil on day 42, and the phagocytic rate of neutrophils of broilers in the whole experiment. We presume that this increase may also be attributed to T cell depletion in the peripheral blood immune system. Numerous studies have shown that ORI has an anti-inflammatory effect. ORI decreases systemic inflammation responses and the increase of phagocytosis, and may be a potential potentially novel therapeutic agent for the treatment of inflammatory disease.

The cytokine level in serum is an important index for humoral immunity in poultry. By now, it is generally known that Th_1_ cells excrete proinflammatory cytokines, such as TNF-α, IL-2 and IL-12, however Th_2_ cells secrete anti-inflammatory cytokines, such as IL-4, IL-5, IL-6, and IL-10 [15]. In the present study, our findings show that serum TNF-α, IL-2 (Th_1_) and IL-4 (Th_2_) in the broilers receiving 50, 80, and 100 mg/kg of ORI were significantly lower than those in control broilers, indicating that ORI could regulate humoral immunity of broilers. In birds, few studies have investigated the immunostimulatory effect of ORI. About our present results, the lower cytokines levels produced in ORI-treated broilers is likely related to ORI’s anti-inflammatory potential through the repression of T-cell immune responses [6]. Our results are similar to previous studies and further indicate that certain terpenoid compounds may play their anti-inflammatory effects through the regulation of Th_1_/Th_2_ cytokine secretion levels or repression of T-cell immune responses. We assume that ORI might regulate the differentiation of Th cells; however, future animal and laboratory tests should be conducted to verify potential mechanisms of action.

## Conclusions

Based on the results of the present work, it was concluded that diet inclusion of ORI increases the relative weight of spleens and bursa, the number of proliferation peripheral blood T and B lymphocytes, and the phagocytic rate of neutrophils, it may also stimulate IL-2, IL-4 and TNF-a production in broilers. Therefore, it could be concluded that dietary use of ORI as a feed additive may improve the immune function and disease resistance ability in broiler production. Further research is needed to understand and clarify the possible mechanisms involved.

## Abbreviations

FCR: Feed conversion ratio
FI: Feed intake
IL-2: Interleukin-2
IL-4: Interleukin-2
MTT: 3-(4,5-dimethylthiazol-2-yl)-2,5-diphenyltetrazolium bromide
NETs: Neutrophil extracellular traps
ORI: Oridonin
PI: Phagocytosis index
PP: Phagocytosis percentage
TNF-a: Tumor necrosis factor-a
WG: Weight gain
